# In Vitro Antioxidant Properties Evaluation of 10 Iranian Medicinal Plants by Different Methods

**DOI:** 10.5812/ircmj.1408

**Published:** 2012-12-06

**Authors:** Soheila Moein, Mahmoodreza Moein, Mohammad Javad Khoshnoud, Tahereh Kalanteri

**Affiliations:** 1 Department of Biochemistry, Faculty of Medicine and Research Center for Molecular Medicine, Hormozgan University of Medical Sciences, Bandar Abbas, IR Iran; 2 Medicinal Plants Research Center and Department of Pharmacognosy, Faculty of Pharmacy, Shiraz University of Medical Sciences, Shiraz, IR Iran; 3 Pharmaceutical Sciences Research Center and Department of Toxicology and Pharmacology, Faculty of Pharmacy, Shiraz University of Medical Sciences, Shiraz, IR Iran

**Keywords:** Phenolic Compounds, Radical Scavenging, Power

## Abstract

**Background:**

There is an interest in finding new and safe antioxidants from natural sources such as medicinal plants.

**Objectives:**

The aim of this study was to evaluate the antioxidant activity of ten Iranian medicinal plants extracts.

**Materials and Methods:**

For antioxidant activity, the radical scavenging activity, reducing power and phenolic contents of ethanol plant extracts were determined. Gallic acid was used as standard reference with well-documented antioxidant activity.

**Results:**

The highest antioxidant activity in terms of DPPH radical scavenging was found in *Verbascum sinuatum L*. *Var* (VS) with an IC50 equal to 263.52 ± 5.981 μg/ml and *Rosa damascena Mill* (RD) with and IC50 equal to 287.9 ± 5.675 μg/ml that are higher than gallic acid (IC50 = 25.32 ± 5.593 μg/ml). The highest antioxidant activity in terms of ferric reducing capacity was also found in *Verbascum sinuatum L*. *Var* extracts (in 85.08 ± 8.66 μg/ml concentration with absorbance 0.5). Also, this extract contains the highest phenolic compounds (8.53 ± 0.11 mg/g).

**Conclusion:**

In this study, *Verbascum sinuatum L*. *Var* contains the highest level of phenolic compounds may be contribute to higher free radical scavenging activity and reducing power in comparison to the other plant extracts. Therefore this plant is a good candidate as natural antioxidant.

## 1. Background 

Synthetic antioxidants are used as food additives and prevent food from deteriorating at legal limits (1). Prevention of free radicals formation and other oxidant substances in food have been the purpose of many research and researches attempt to identify antioxidant compounds in natural substances (2, 3). Today’s, much attention has been focused on the use of natural antioxidant to protect from damage due to free radicals (4). Antioxidants play an important role in inhibition and radical scavenging, thus providing protection against diseases. Antioxidants inhibit many oxidation reactions caused by free radicals such as singlet oxygen, superoxide radicals, proxy radicals, hydroxyl radicals and proxy nitrate (5). The methods mostly used to determine antioxidant activities are divided into two main groups. First method based on one electron transfer reaction, which a change in color occurred when the oxidant compound was reduced. Second method based on one hydrogen transfer reaction and competition between antioxidant and substrate (probe) for free radicals was occurred (6). A great number of medicinal plants contain chemical compounds which possess antioxidant activities. Natural antioxidants are mainly phenolic compounds that may exit in all parts of the plants 4 and possess antioxidant activities.

## 2. Objectives 

The aim of the present study was to investigate the antioxidant activities of ten medicinal plants which grow in Fars province (south western of Iran). These plants include *Verbascum sinuatum L*. (VS), *Rosa damascene Mill* (RD), *Tripleurospermum disiforme* (TD), *Phlomis olivieri Benth* (PO), *Stachys pilifera Benth* (SP), *Chenopodium foliosum* (moench) Aschers (CF), *Onosma rostellatum Lehm* (OR), *Ziziphora tenuir L* (ZT), *Salvia macrosiphon Boiss* (SM), *Centaurea depressa* M.B (CD). For evaluation the antioxidant activities, DPPH radical scavenging, reducing power and phenolic content of these extracts were detected.

## 3. Materials and Methods

**Figure 1:**
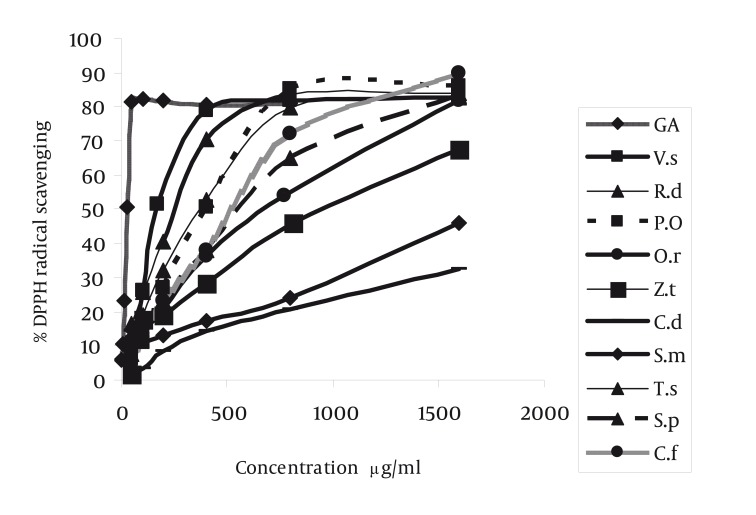
Radical (DPPH) Scavenging Activity of Plants Extracts Compared With Standard gallic Acid

The plant materials were collected according to Table 1 and identified by Dr. A. Khosravi (Department of Biology, Shiraz University and Miss S. Khademian (Department of Pharmacognosy, Faculty of Pharmacy, Shiraz University of Medical Sciences). The voucher specimens were deposited in herbarium of Department of Pharmacognosy, Faculty of Pharmacy, Shiraz University of Medical Sciences (Table 1). Aerial parts of plants (40 g) were macerated in ethanol (96%) for 48 h. The extracts were filtered and concentrated under reduced pressure in 40°C and the extracts were further diluted by methanol (50-800 µg/ml). A series concentration of gallic acid was made (3.125- 800 µg/ml) as standard. The antioxidant activities of all crude extracts were measured by radical scavenging ability using DPPH radical. An ethanolic solution (20 µL) of the samples at various concentrations (50-800 µg/ml) were added to 200 µL of ethanol solution of DPPH (100 mM) in microplate (7). Negative controls were prepared with 20 µL ethanol and 200 µL DPPH in triplicate. Each well contained 200 µL solution of DPPH (100 mM) and 20 µL of extract. The micro plates were incubated at 25°C for 30 min and the absorbance was measured at 492 nm using a micro plate reader (Stat Fax® 2100, Awareness Technology, Inc, USA). Gallic acid was used as an antioxidant standard. The obtained data were used to determine the concentration of the samples required to scavenge 50% of the DPPH free radicals (IC50). The percent inhibition was plotted against the concentrations of extract and the IC50 was obtained from the fitted linear curve, curve expert Ver 1.3. The results were expressed as the mean ± SD of three replicates (Figure 1). The concentrations of Gallic acid as antioxidant standard were (3.125-800 µg/ml). All determinations were performed in triplicate (n =3).

**Table 1:** Collection Locality, Collection Time and Voucher Number of Studied Plants Growing in Fars Province

Scientific Name	Genus	Collection Time	Collection Locality	Herb Number	Extract Yield, %
*Verbascum Sinuatum L. var*.	Scrophulariaceae	Agust 2005	Kakan	Pm. 21	12.33
*Rosa Damascena Mill*.	Rosaceae	Agust 2007	Kamfirooz	Pm.18	19.075
*Ziziphora tenuir L*.	Lamiaceae	Agust 2007	Safa shahr	Pm.17	5.25
*Stachys Pilifera Benth*.	Lamiaceae	Agust 2006	Kamfirooz Marvdasht	500	17.13
*Tripleurospermum Disiforme*	Asteraceae	May 2006	Kakan	511	6.93
*Centaurea Depressa M.B*.	Asteraceae	Agust 2007	Kamfirooz Marvdasht	Pm.19	5.3
*Salvia Macrosiphon Boiss*.	Lamiaceae	Agust 2007	Pasargad	305	6.7
*Chenopodium Foliosum (moench) Aschers*.	Chenopodiaceae	Agust 2006	Safa shahr	261	10.35
*Phlomis olivieri Benth*.	Lamiaceae	Agust 2006	Kamfirooz Marvdasht	Pm.16	6.77
*Onosma Rostellatum Lehm*.	Boraginaceae	May 2007	Marvdasht	Pm.20	9.55

The reductive potential of plant crude extracts was determined according to the method described by Souri (8). The different concentrations of plant extracts were made (50- 800 µg/ml) in 0.2 M phosphate buffer pH 6.6 containing 1% ferrocyanate. The mixture was incubated at 50°C a portion (2.5 ml) of trichloroacetic acid (10 % w/v) was added to the mixture, which was then centrifuged at 3000 g for 10 min. The upper layer was separated and mixed with 2.5 ml of distilled water containing 0.5 ml of ferric chloride 1%. The absorbance of this mixture was measured at 700 nm using a UV-Vis spectrophotometer (T80 plus, PG Instrument, UK). The intensity in absorbance showed the antioxidant activities of plant extracts (8). The concentrations of gallic acid as antioxidant standard were (3.125-800 µg/ml). All determinations were performed in triplicate (n = 3). The content of total phenolic compounds in ethanol extracts was determined by a modification in folin-ciocalteu method (5). For the preparation of calibration curve, 0.5 ml aliquots of 0.024, 0.075, 0.105 and 0.3 mg/ml ethanol gallic acid solutions were mixed with 2.5 ml folin-ciocalteu reagent (diluted ten –fold) and 2 ml (75 g/l) sodium carbonate. The contents were mixed and allowed to stand for 30 min.

Absorption at 765 nm was measured in a UV-Vis spectrophotometer (T80 plus, PG Instrument, UK). Half of one ml plant extracts (10 g/l) was mixed with the same reagents as described above, and after 1 hour the absorption was measured for the determination of plant phenolics. All determinations were performed in triplicate. Total content of phenolic compounds in plant ethanol extracts in gallic acid equivalents (GAE) was calculated by the following formula:

C= c.v/m

Where: C is the total content of phenolic compounds, mg/g plant extract, in GAE; c is the concentration of gallic acid established from the calibration curve, mg/ml; v is the volume of extract, ml; m is the weight of pure plant ethanol extract, g (5). All determinations were performed in triplicate (n = 3). Data expressed as mean ± standard deviation (SD). The IC50 was calculated by using Curve Expert Version 1.3. The data were analyzed for statistical significance using one way ANOVA followed by tukey posttest in SPSS ver. 16. *P* value less than 0.05 was considered significant. In statistical analysis, variables (including IC50 of plant extract, reducing power and total phenolic content) were compared among different plant extracts. The IC50 is the concentration of plant extract which scavenged 50% of the DPPH free radical. In reducing power, the concentration of plant extract, which showed OD = 0.5 was calculated. For determination phenolic compounds concentration of plant extracts following formula was used.

## 4. Results

**Figure 2:**
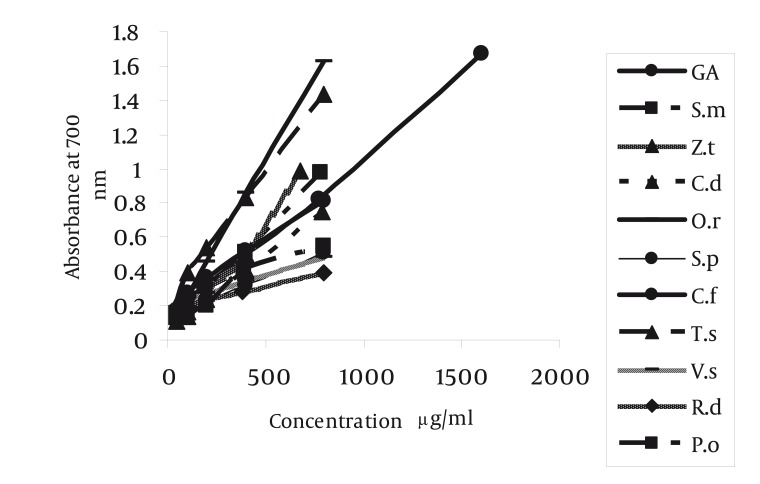
Reducing Power of Plants Extracts Compared with Standard Gallic Acid

**Figure 3:**
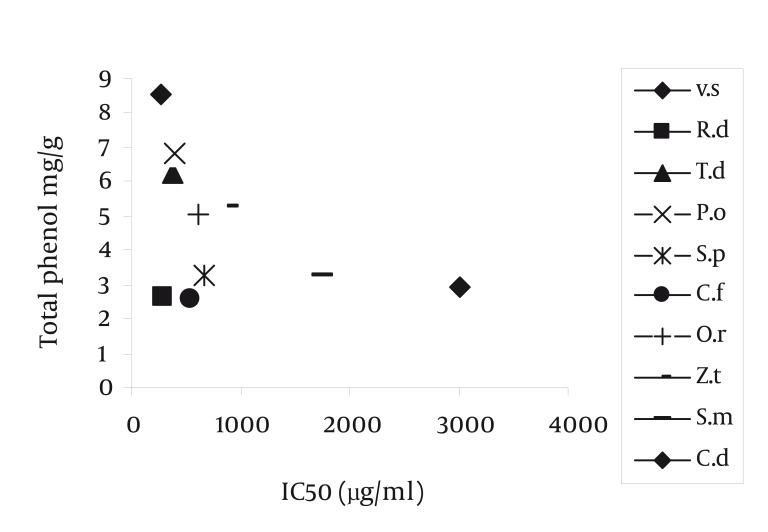
Relationship Between IC50 and Total Phenols of Plant Extracts

Radical scavenging capacity increased as extracts concentrations raised from 50 to 1600 µg/ml. The IC50 of gallic acid was lower than the IC50. of plants extracts. In other words, the IC50 of plant extracts decreased in the following order: gallic acid < VS = RD< TD = PO < SP =CF < OR< ZT < SM = CD extract (Table 2) (P < 0.001). The highest and lowest reducing power was observed in VS and CD extracts respectively. Thus, the decreasing order of reducing power of the extracts was gallic acid > VS > PO > SP > RD > TD > OR> ZT > SM > CF > CD extract. For comparison the reducing power of plant extracts, the concentration which showed absorption of 0.5 was determined (Table 1, Figure 2). On the basis of these results, the reducing powers of all extracts are less than gallic acid. Phenolic contents, expressed as gallic acid equivalents, varied from 2.6 ± 0.02 to 8.53 ± 0.11 mg/g (Table 2). The highest amount of phenolic compounds was found in VS extract. Therefore, the phenolic contents of the extracts increased in the following order VS > PO = TD > ZT = OR > SM = CD = SP = RD = CF extract, respectively. VS extract possess the highest radical scavenging activity and the amount of phenolic compounds (Figure 3, Table 2).

**Table 2:** DPPH Radical Scavenging, Reducing Power and Phenolic

Sample a	IC50 (µg/ml), Mean ± SD	Extract Concentration with Absorption 0.5 µg/ml, Mean ± SD	Total phenolic content mg/g, Mean ± SD
*Gallic aicd*	25.32 ± 5.59 A	10 ± 0.26	NA b
*Verbascum Sinuatum L. var.*	263.52 ± 5.98 B	85.08 ± 8.66	8.53 ± 0.11 A
*Rosa damascena Mill.*	287.9 ± 5.68 B	166.39 ± 10.13	2.63 ± 0.16 B
*Ziziphora tenuir L.*	884.4 ± 5.47 C	389.63 ± 6.65	5.27 ± 0.09 D
*Stachys pilifera Benth.*	535.4 ± 4.84 D	145.39 ± 0.19	2.76 ± 0.02 B
*Tripleurospermum Disiforme*	374.95 ± 6.26 E	197.44 ± 5.64	6.66 ± 0.19 E
*Centaurea Depressa M.B.*	> 1600 NA	513.05 ± 21.3	2.91 ± 1.02 NA
*Salvia Macrosiphon Boiss.*	> 1600 NA	392.79 ± 9.38	3.27 ± 0.02 NA
*Chenopodium Foliosum (moench) Aschers.*	546.88 ± 5.90 D	408 ± 5.44	2.6 ± 0.02 B
*Phlomis Olivieri Benth.*	397.26 ± 6.61 E	134.33 ± 8.16	6.85 ± 0.27 E
*Onosma Rostellatum Lehm.*	605.96 ± 5.41 F	219.98 ± 2.64	5.06 ± 0.12 D

^a^Concentration of plant extract in absorbance 0.5. Statistical differences showed as alphabetic letters. The same alphabetic letters implied there are not any statistical differences P > 0.05 and different letters represented statistical differences P < 0.05

^b^Abbreviations: NA, Non Active

## 5. Discussion

It is found a significant relation between radical scavenging and phenolic content of extracts, exception CF, SP and RD extracts.

**Figure 4:**
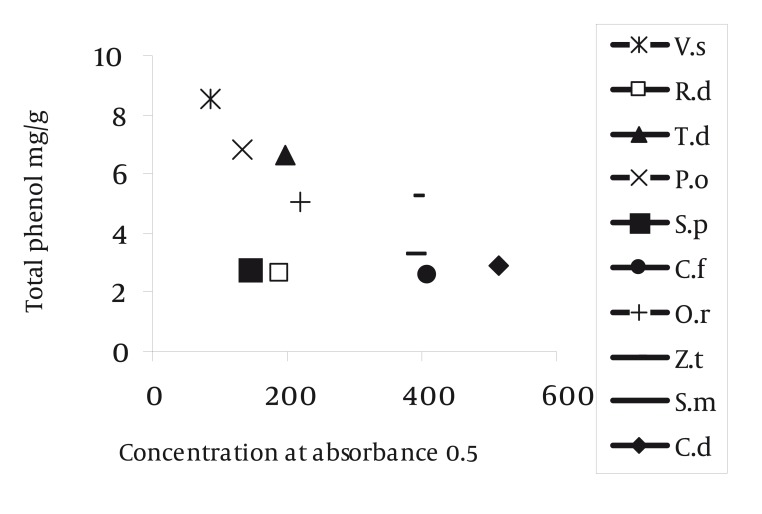
Relationship Between Absorbance 0.5 in Reducing Power and Total Phenols of Plant Extracts

**Figure 5:**
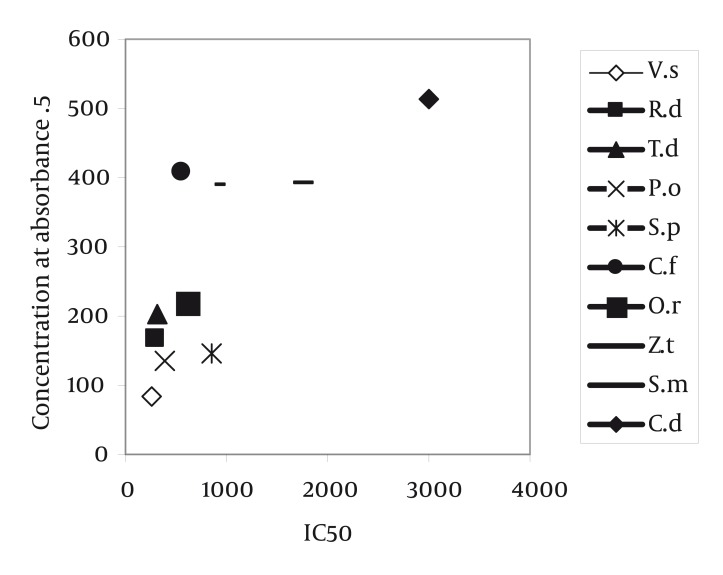
Relationship Between Absorbance 0.5 in Reducing Power and IC50 of Plant Extracts

It seems that, in CF, SP and RD extracts non-phenolic compounds involve in DPPH radical scavenging (Figure 3). These results showed that the IC50 of all plant extracts were higher than gallic acid (Table 2). The IC50 of VS extract (263.52 ± 5.98 µg/ml) was less than the other plant extracts. The IC50 of SM (1600 µg/ml) and CD (1600 µg/ml) extracts were higher than the other plant extracts. In other words, these two extracts could not effectively scavenge DPPH radical (Table 2). In other researches, it was reported that other species of Verbascum, like Verbascum lasianthum, Verbascum cilicium, Verbascum salvifolium, Verbascum macrurum and Verbascum pterocalycinum extracts could scavenge DPPH free radicals (9, 10). Also, other species of Phlomis, like Phlomis physocalyx (11), Phlomis persica, (12), Phlomis samia, (11), Phlomis monocephala and Phlomis carica (13) possess this property. The IC50 of Phlomis persica (169.8 ± 5) is less than the IC50 of PO extract (397.26 ± 6.61) (12). This means that the radical scavenging activity of Phlomis persica is more than PO extract. In reducing power assay, the antioxidant compounds cause reduction in Fe+3 by donation one electron. The absorption of Fe +2 is determined at 700 nm and the higher absorption shows more reducing power. This property in VS extract was higher (85.05 ± 8.66 µg/ml) and in CD extract (513.05 ± 21.3 µg/ml) was lower than the other extracts, respectively. The antioxidant potential and phenolic diversity in many plants have been previously reported, such as persimmon, (14) sea buckthorn (15) etc. In present study, the amount of phenolic compounds, measured by folin-ciocalteu reagent, was expressed as gallic acid equivalent. Differences between phenolic compounds arise from the specific structure of each compound (number of OH groups, side chain on benzoic acid, etc.) instead of the phenolic family (16). Phenolic compounds by donation of one H+ cause scavenging free radicals and inhibition of macromolecules damage. Other research, reported that the total phenolic compounds of Salvia mirzayanii ethyl acetate fraction is high (49.23 ± 3.4 mg/g) (7). In other words by fractionation of plant extract, it is possible to obtain compounds with more phenolic compounds. There is a positive correlation between DPPH radical scavenging and phenolic content of plant extracts (17). In other words, in these extracts, phenolic compounds may be responsible for a part of radical scavenging activity (18). In other researches, a moderate correlation was found between DPPH radical scavenging and phenolic content in Iranian plant extract, Salvia mirzayanii (7). Some researchers were not found any correlation between free radical scavenging and phenolic compounds (19). Relationship between reducing power and the amount of phenolic compounds (Figure 4) imply that VS extract possess the highest reducing power and the amount of phenolic compounds. In this research, we found a positive relation between reducing power and the amount of phenolic compounds, exception SP and RD extracts (Table 1). In these two extracts, may be non-phenolic compounds possess antioxidant activities (Figure 3). Also, other researchers did not find a significant correlation between antioxidant activity and phenolic content of the studied plants and in this situation phenolic content could not be a good marker of antioxidant capacity (6). In other articles, the antioxidant properties of different species of Rosa like Rosa rugosa and Rosa roxburghii is reported (20). Also, other species of Onosma like Onosma hispida, Onosma argentatum (21) and other species of Salvia like Salvia officinalis, (22) Salvia mirzayanii (7) showed this activity. Comparison between OR and ZT extracts show that these two extracts possess similar phenolic contents (Table 2) but antioxidant activities of OR is more than ZT (*P* < 0.05). May be other compounds, include non-phenolic compounds of OR extract involve in antioxidant activities. A relationship between reducing power and radical scavenging (IC50) is observed in OR, OP, VS and ZT extracts. This relation was not always observed, i.e. the IC50 of RD is less than PO extract, but its reducing power is less than PO extracts (Figure 5). Totally, VS extract possess the highest antioxidant activity and phenolic compounds. The anti-inflammatory effect of this extract may be attributed to its antioxidant activity. The second extract which possesses more antioxidant capacity is RD extract which prevents cardiovascular disease. Also this extract, possesses anti-aging compounds. These properties of RD extract may be attributed to its antioxidant activity. Natural antioxidants of studied plants may be as potent candidates in prevention of diseases arise from free radicals such as cancer, cardiovascular disease, diabetes and aging process. Anyway, plants compounds have important applications effects in therapy with fewer side effects (23).
